# Metformin Monotherapy Downregulates Diabetes-Associated Inflammatory Status and Impacts on Mortality

**DOI:** 10.3389/fphys.2019.00572

**Published:** 2019-05-21

**Authors:** Anteneh Mehari Tizazu, Ma Shwe Zin Nyunt, Olivier Cexus, Koolarina Suku, Esther Mok, Chin Hui Xian, Joni Chong, Crystal Tan, Wilson How, Sandra Hubert, Emilie Combet, Tamas Fulop, Tze Pin Ng, Anis Larbi

**Affiliations:** ^1^Singapore Immunology Network, Agency for Science, Technology and Research (A*STAR), Singapore, Singapore; ^2^Department of Microbiology and Immunology, Yong Loo Lin School of Medicine, National University of Singapore, Singapore, Singapore; ^3^Department of Psychological Medicine, Yong Loo Lin School of Medicine, National University of Singapore, Singapore, Singapore; ^4^Human Nutrition, School of Medicine, College of Medical, Veterinary and Life Sciences, University of Glasgow, Glasgow, United Kingdom; ^5^Research Center on Aging, Graduate Program in Immunology, Faculty of Medicine and Health Sciences, University of Sherbrooke, Sherbrooke, QC, Canada; ^6^Department of Biology, Faculty of Sciences, Tunis El Manar University, Tunis, Tunisia

**Keywords:** aging, diabetes, chronic inflammation, metabolic syndrome, insulin resistance, metformin

## Abstract

Aging is the main risk factor for developing diabetes and other age-related diseases. One of the most common features of age-related comorbidities is the presence of low-grade chronic inflammation. This is also the case of metabolic syndrome and diabetes. At the subclinical level, a pro-inflammatory phenotype was shown to be associated with Type-2 diabetes mellitus (T2DM). This low to mid-grade inflammation is also present in elderly individuals and has been termed inflammaging. Whether inflammation is a component of aging or exclusively associated with age-related diseases in not entirely known. We used clinical data and biological readouts in a group of individuals stratified by age, diabetes status and comorbidities to investigate this aspect. While aging is the main predisposing factor for several diseases there is a concomitant increased level of pro-inflammatory cytokines. DM patients show an increased level of sTNFRll, sICAM-1, and TIMP-1 when compared to Healthy, Non-DM and Pre-DM individuals. These inflammatory molecules are also associated with insulin resistance and metabolic syndrome in Non-DM and pre-DM individuals. We also show that metformin monotherapy was associated with significantly lower levels of inflammatory molecules, like TNFα, sTNFRI, and sTNFRII, when compared to other monotherapies. Longitudinal follow up indicates a higher proportion of death occurs in individuals taking other monotherapies compared to metformin monotherapy. Together our finding shows that chronic inflammation is present in healthy elderly individuals and exacerbated with diabetes patients. Likewise, metformin could help target age-related chronic inflammation in general, and reduce the predisposition to comorbidities and mortality.

## Introduction

Population aging has become a concern for both developed and developing nations. The number of individuals over the age of 60 was 900 million in 2015 and this number is expected to rise to 2 billion by the year 2050, of which 80% of this population will be living in middle and low-income countries. This demographic change is a current challenge for developed nations like Japan where 30% of the population is over the age of 60 years (World Health Organization [WHO], 2015). Aging is the main risk factor for developing an age-related disease such as cancer, Alzheimer’s disease and cardiovascular diseases (World Health Organization [WHO], 2011). Diabetes is one of the common diseases associated with aging and its risk increases with age (Nguyen et al., 2012). Type-2 Diabetes mellitus (T2DM) has a worldwide impact, as the disease has contributed to 1.5 million deaths in 2012. The number of individuals having diabetes has increased from 180 million in 1980 to 422 million in 2014 and its prevalence has almost doubled from 4.7% in 1980 to 8.5% in 2014 among adult population (World Health Organization [WHO], 2016). One of the main issues with DM is the collateral damages as shown by the increased prevalence of conditions impairing kidney function, cardiovascular health, eye sight and others.

One of the most common features of most age-related comorbidities including diabetes and its complications is the presence of low-grade chronic inflammation (Michaud et al., 2013). The underlying sources for this inflammation include chronic infection, immunosenescence and obesity (Pawelec et al., 2014). Not enough has been done to identify a common mechanism favoring the onset of age-related chronic inflammation and its consequence, rather much focus, and resources have been devoted to tackle individual age-related comorbidities (Goldberg and Dixit, 2015). Chronic inflammation is getting more attention as a possible player in many metabolic conditions such as for insulin resistance and this could impact future treatments. For instance, pro-inflammatory cytokines like TNFα (Tumor Necrosis Factor α) which concomitantly increase with adipose tissue accumulation can have a systemic metabolic effect (Feinstein et al., 1993; Hotamisligil et al., 1993).

As the aging process depends on many factors from genetics to environment, identifying and targeting different markers of aging vs. age-related diseases is crucial to identify targets that promote healthspan. Pharmacological therapy using known and safe drugs like metformin has become one option (Valencia et al., 2017). Metformin is a glucose-lowering drug that has been used for more than 60 years (Bailey, 2017). This biguanide is the first-line treatment against DM but possesses diverse pleiotropic properties. Animal and clinical studies have suggested other beneficial effects of metformin besides glucose control. Metformin decreases inflammatory cytokines, such as TNFα, interleukin (IL)-6, and IL-1, and the inflammatory response of macrophages and induces the production of anti-inflammatory cytokines such as IL-4 and IL-10 (Hyun et al., 2013; Cameron et al., 2016). It also has a beneficial role in preventing DM (Diabetes Prevention Program Research Group, 2015), decreases risk of macrovascular disease and weight gain (Kooy et al., 2009), anti-depressant effect (Guo et al., 2014), and decreases risk of cognitive impairment (Ng et al., 2014). Beside preventing age-related comorbidities metformin treatment increase healthy lifespan in mice (Anisimov et al., 2011; Martin-Montalvo et al., 2013) and in *Caenorhabditis elegans* (Cabreiro et al., 2013; De Haes et al., 2014). This cumulative data on the beneficial use of metformin has led to the upcoming studies like the Veterans Affairs’ Investigation of Metformin in Pre-Diabetes on Atherosclerotic Cardiovascular OuTcomes (NCT02915198) which will assess the role of metformin in non-diabetes individuals.

Aging and DM patients are both associated with increased inflammation. As aging trajectories and DM management can be quite heterogeneous, dissecting the inflammatory markers in clinically stratified cohorts (by age and health status) would help identifying the impact of age, disease and treatment in the control of inflammation. Thus, our aim in this study was to assess inflammation in participants of the Singapore Longitudinal Aging Study stratified by age, diabetes status, medication and taking other comorbidities in consideration. The beneficial role of metformin treatment was tested for soft (inflammation) and hard outcomes (mortality). Our data suggest that DM patients taking metformin are significantly advantaged at the inflammatory level and larger studies should confirm data from our pilot study that metformin may ultimately reduce mortality in DM patients.

## Materials and Methods

### Study Subjects

The elderly individuals of this study are part of the Singapore Longitudinal Aging Study 2 (SLAS-2), which is an undergoing population-based cohort intended to study the biology of aging among Singaporean elderly individuals above the age of 55 years old. The SLAS-2 study measures different parameters of 3270 elderly Singaporean. The participants were recruited by a door to door census and only volunteer individuals participated in the study. The response rate to participate in the study was 78.5%. Volunteer participants completed a range of tests and answered a series of interview questions within 5–6 interview sessions. The interview includes socio-demographic data (age, gender, ethnicity) medical history (hospitalization, medical status, types of medication), physical health (regular exercise, consumption of alcohol, cigarette smoking habit) and nutritional intake. Whereas tests like Boston Naming Test (BNT) and the revised Brief Visuospatial Memory Test (BVMT-R) were used to assess cognitive function. Standard physical examination (height, weight, waist and hip ratio, body mass index) and tests like Performance-Oriented Mobility Assessment (POMA), hand grip strength, knee extension test were used to assess the function of the body. Blood analysis (fasting blood glucose, blood count, hematocrit level, albumin, creatinine, estimated glomerular filtration rate) was done by taking a blood sample. Elderly individuals physically incapable to participate in the study and those individuals with mental disorders that could not give informed consent were excluded from the study. The study was approved by the National University of Singapore Institutional Review Board, and all participants provided written informed consent. The young control individuals were recruited from the National University of Singapore and any young individual with chronic disease, taking medication or recently hospitalized was excluded from the study. The detailed procedure and characteristic of the study cohort have been previously described (Ng et al., 2009; Lu et al., 2016; Valenzuela et al., 2017).

### Operational Terms

Here for the purpose of this paper we use the following terms to refer to the specific group, Young refers to individuals, age between 18 and 29 years, who have no comorbidity and do not take any medication, Healthy refers to elderly individuals age range of 55–94 years old, who have no comorbidity and do not take any medication. Non-Diabetes (Non-DM) represent elderly individuals age range 55–94 years old, who are non-diabetes but have at least one comorbidity and take medication for a specific disease or diseases. Pre-Diabetes (Pre-DM) represent elderly individuals, age range 55–94 years old, who have fasting blood glucose between 5.6 and 6.9 mmol/L with no distinction based on co-morbidity. Diabetes (DM) represents elderly individuals age range 55–94 years old, those who have a confirmed case of diabetes and taking the corresponding medication.

### Exclusion Criteria

Diabetes patients with no clear anti-diabetes treatment were not included in the study. Likewise, from the healthy group individuals those under cholesterol-lower medication were excluded despite the apparent normal cholesterol levels. Finally, any individual that has a specific disease like hypertension and heart disease but that does not take medication for the specific disease was removed from the analysis. Individuals with severe physical disabilities or severe to moderate cognitive impairment (MMSE <19), were excluded from the analysis.

### Serology

Blood samples were collected from overnight fasting individuals in BD Vacutainer^®^ CPT^TM^ Cell Preparation tubes with Sodium Citrate (BD Biosciences, San Jose, CA, United States) and centrifuged at 300 rcffor 20 min. Plasma samples were collected and stored at −80°C. Multiplex technology (Millipore Corp., Billerica, MA, United States) was used according to manufacturer’s instruction to measure 102 different human chemokines and cytokines in young and old individuals. After overnight incubation, the plates were read on a Flexmap 3D instrument (Luminex Corporation, Austin, TX, United States), and data were analyzed using Bioplex Manager 6.0 software (Bio-Rad Laboratories, Hercules, CA, United States).

### Fructosamine Measurement

The level of fructosamine was measured using nitro blue tetrazolium (NBT) assay, performed in microplates. Twenty five microliters of the sample was added to sodium carbonate buffer (100 μl and 100 mM, pH 10.8) with nitro blue tetrazolium (0.25 mM). Microplates were incubated for 15 min at 37°C and measured spectrophotometrically against controls at 550 nm after 10 and 15 min of incubation. The difference between the two readings was used to calculate the concentration. The fructosamine analog DMF (dimethylformamide) was used as a standard. All fructosamine measurements were performed in duplicate. Standards and NBT reagent were made fresh every week and stored at −20 and 4°C, respectively (Baker et al., 1993; Vlassopoulos et al., 2013).

### Hematological Profile and General Blood Profiling

White blood cell count, lymphocyte count, eosinophil count, monocyte count, basophil count, RBC count, platelet count, hematocrit, MPV, RBC distribution width, mean cell hemoglobin (MCH), MCHC, and MCV were obtained using a Beckman Coulter Hematology Analyzer (Beckman Coulter, CA, United States). Serum levels of glucose, creatinine, estimated glomerular filtration rate (eGFR), triglyceride, total cholesterol, HDL cholesterol, LDLs, cholesterol and ion concentration were measured using standard laboratory procedures and techniques at the National University Hospital Reference Laboratory (NUHS, Singapore). HOMA2-IR C-peptide (Homeostasis Model Assessment-Insulin Resistance C-peptide) was calculated using a fasting blood glucose and C-peptide level using software developed by Diabetes Trials Unit, University of Oxford (Diabetes Trials Unit, 2017).

### Statistical Analysis

Data analysis was done using R Commander package from R software (V3.3.2) and GraphPad Prism (V7.03). The data was cleaned using Excel spreadsheet and subjects with missing variables or those individuals with incomplete information were removed from the analysis. The distribution of the data was checked using Shapiro–Wilk test using R software (V3.3.2) and means and Standard Deviation (SD) or median and interquartile ranges were used to describe contentious data with or without normal distribution, respectively, and percentage was used for categorical data set. χ^2^ tests and Kruskal–Wallis tests were used to compare categorical variables and quantitative variables between groups, respectively. Principal Component Analysis (PCA) was done by first converting the data set into a logarithmic format (base 10 log was used) and using Facto minor package of R software. Spearman correlation coefficient was used to correlate measured cytokines with one another and with insulin resistance. The ggplot2 package was used to generate the volcano plots. A statistical test was done by first running an unbiased comparison of all measured chemokines and cytokines between young and the four different old groups (Healthy, Non-DM, Pre-DM, and DM) using Mann–Whitney *U*-test, we then applied Bonferroni multiple testing correction (0.05/102 = 4.9^–04^) and set the *P*-value 4.9^–04^ as a cut of *P*-value and molecules that showed a *P*-value less than 4.9^–04^ were considered as statistically significant. In similar fashion, we used Kruskal–Wallis test to compare all measured molecules in an unbiased manner between the four different old groups (Healthy, Non-DM, Pre-DM and DM) and then applied Bonferroni multiple testing correction and molecules that showed a *P*-value less than 4.9^–04^ were considered as statistically significant. For further downstream comparison of those molecules that pass the Bonferroni multiple testing correction a *P*-value <0.05 with a two-side distribution was considered as statistically significant. The survival data was analyzed using GraphPad prism and fisher exact test or χ^2^ test was used depending the number of cases in each group. The odds ratio and confidence interval was also generated using GraphPad prism.

## Results

### Socio-Demographic Characteristics and Clinical Determinants of DM

**FIGURE 1:**
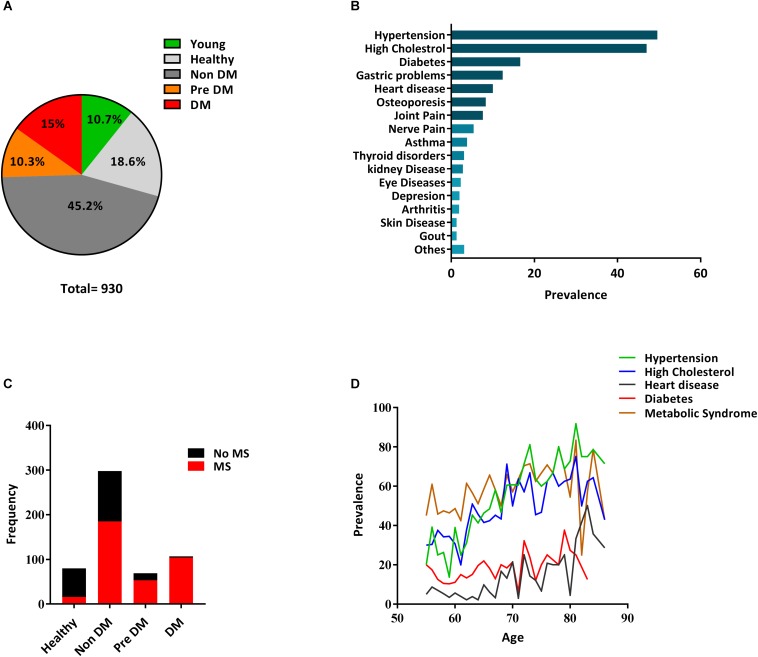
Distribution of diabetes and other diseases in the SLAS cohort. **(A)** A pie chart indicating the percentage of the individual in each group. **(B)** Prevalence of confirmed medical conditions in the SLAS cohort. Others indicate [skin disease (1.67%), Gout (1.67%) chronic obstructive Pulmonary Disease (0.76%), Parkinson disease (0.3%), Anemia (1.2%), and Prostate cancer (1.07%)]. **(C)** Cases of metabolic syndrome in the four groups using the updated NCEPATP III [National Cholesterol Education Program (NCEP) Adult Treatment Panel III (ATP III)] criteria. The Proportion of Metabolic syndrome within Healthy, Non-DM, Pre DM, and DM group is 20, 62, 76.8, and 97.2%, respectively. **(D)** Age-dependent Prevalence of Diabetes, Hypertension, Heart Disease, High Cholesterol, and Metabolic Syndrome in the SLAS cohort.

In this study we have stratified the elderly group in order to identify the healthiest vs. individuals with diabetes with/without comorbidities. We arbitrary used the term “healthy aging” for elderly individuals with no major morbidity (Lara et al., 2015) and “unhealthy aging” for elderly individuals with comorbidities. We used medical history, clinical data, structured questionnaires, and measured biological parameters to subdivide our cohort (*n* = 930) into four groups as, confirmed cases of DM patients (DM), non diabetes individuals having no comorbidity (Healthy), non diabetes individuals having at least one comorbidity (Non-DM), and pre-diabetes individuals with fasting blood glucose between 5.6 and 6.9 mmol/L (Pre-DM) as detailed in the Section “Materials and Methods” of the manuscript (Figure 1A). A group of young individuals with no history of diabetes was also included.

Hypertension (49.6%), hypercholesterolemia (47%), and DM (16.6%) represent the three prevalent conditions in our cohort (Figure 1B and Supplementary Table 1) and the occurrence of this disease gradually increase along with age (Figure 1D). Our analysis also demonstrates the incremental prevalence of metabolic syndrome (MS) from Healthy to Non-DM, Pre-DM, and DM individuals (Figure 1C). We then matched age, sex and BMI in the four groups and found the genetic background to be associated with differential prevalence. Indians have a higher prevalence of diabetes (*P* < 0.05) compared to both Chinese and Malays (Table 1) which is concurrent with a previous report in Singapore (Chiang et al., 2011). There was no significant difference between the four groups on smoking status but significantly low (*P* < 0.05) ApoE-ε4 (Apolipoprotein E-ε4) carrier status was found in DM groups compared to the three other old groups (Table 1). A high proportion of individuals in the DM group take lipid-lowering drugs which is reflected in the amount of total cholesterol and LDL (Table 2, *P* < 0.001). While HDL levels are lower in the DM group, lipid-lowering drugs tend to improve the LDL/HDL ratio. The liver function tests used, ALT/GPT (Alanine aminotransferase/Glutamic-Pyruvate Transaminase) and AST (Aspartate aminotransferase) do not show a significant difference between the four groups. The hematological parameters show significantly higher (*P* < 0.05) count of circulating WBC, lymphocyte, monocyte, eosinophil, and significantly lower (*P* < 0.05) level of Hematocrit and MCV in DM group compared to the other three old groups (Table 2), also concurring with a previous study (Zhang et al., 2017). This may suggest a dysregulated immune homeostasis with diabetes.

**TABLE 1:** General socio-demographic characteristics.

Characteristic	Healthy (*n* = 81)	Non-DM (*n* = 299)	Pre DM (*n* = 70)	DM (*n* = 108)	χ^2^	*P*-value
Age	65 (62–69)	66 (62–72)	67 (62–72.75)	66 (63–74)	2.5	0.47
Gender, female n (%)	45 (55.6)	187 (62.5)	38 (54.3)	70 (64.8)	3.3	0.35
BMI	24.6 (22.2–26.9)	24.6 (22.3–26.7)	24.9 (23.3–27.4)	25.5 (23–28.4)	7.2	0.064
**Race**						
Chinese	71 (14.8)	265 (55.2)	61 (12.7)	83 (17.3)		
Malay	8 (17)	23 (48.9)	7 (14.9)	10 (20.8)	21.5	0.0014
Indian ¬Δ	2 (6.9)	10 (34.5)	2 (6.9)	15 (51.7)		
Smoking						
Non/EX/smoker (n)	64/10/7	247/31/21	55/8/7	85/13/10	1.55	0.96
Non/EX/smoker (%)	79/12.3/8.6	82.6/10.4/7	78.6/11.4/10	78.7/12/9.3		
ApoE-ε4 carrier, n (%)	11 (14.1)	56 (19.2)	10 (14.5)	6 (5.8)^a^	10.4	0.014
**Glucose metabolism**						
HOMA2-IR C-peptide*	0.73 (0.53–0.9)	0.86 (0.64–1.1)^b^	1 (0.84–1.46)^c,d^	1.27 (0.94–1.90)^a,e,f^	84.5	2.2^–16^
Fasting glucose (mmol/L)	4.8 (4.6–5.1)	4.8 (4.5–5.1)	5.85 (5.7–6.2)^c,d^	7.2 (5.8–8.5)^a,e,f^	296	2.2^–16^
Fructosamine (NBT conc)	0.24 (0.23–0.27)	0.25 (0.23–0.27)	0.27 (0.24–0.28)^c,d^	0.29 (0.26–0.33)^a,e,f^	58.4	1.28^–12^

*HOMA2-IR C-peptide (Homeostasis Model Assessment-Insulin Resistance C-peptide) was calculated using a fasting C-peptide and Fasting glucose level using a software developed by Diabetes Trials Unit, University of Oxford (Diabetes Trials Unit, 2017). The HOMA2-IR is expressed in arbitrary units. For each measured parameter the result in the table indicate a Median and interquartile range (IQR, 25–75th percentile) or number (%) as appropriate. Significant difference (*P* < 0.05) between two groups is indicated by the symbols, ^a^DM vs. Non-DM, ^b^Non-DM vs. Healthy, ^c^Pre-DM vs. Healthy, ^d^Pre-DM vs. Non-DM, ^e^DM vs. Healthy, and ^f^DM vs. Pre-DM. Indian have a significantly higher prevalence of diabetes when compared to Chinese (¬) and Malay (Δ) and no significant difference was observed between Chinese and Malay on the prevalence of diabetes.

**TABLE 2:** Basic clinical of participants.

Characteristic	Healthy (*n* = 81)	Non-DM (*n* = 299)	Pre DM (*n* = 70)	DM (*n* = 108)	χ^2^	*P*-value
**Markers of lipid profile**						
Lipid-lowering drug n (%)	0 (0)	165 (55.2)^d^	22 (31.4)^b,e^	88 (81.5)^a,c,f^		2.20^–16^
Triglycerides (mmol/L)	1.16 (0.89–1.5)	1.24 (0.9–1.6)	1.26 (0.91–1.9)	1.42 (1–1.8)^a,f^	8.1	0.044
Total cholesterol (mmol/L)	5.45 (5–6.1)	4.99 (4.4–5.7)^d^	5.39 (4.7–5.9)^b^	4.33 (3.8 – 5.0)^a,c,f^	72.1	1.51^–15^
HDL-C (mmol/L)	1.41 (1.3–1.6)	1.40 (1.2–1.6)	1.36 (1–1.6)	1.19 (1.0–1.4)^a,c,f^	7.89	3.8^–06^
LDL-Cholesterol (mmol/L)	3.4 (2.9–4)	2.9 (2.4–3.5)^d^	3.2 (2.6–3.8)^b^	2.35 (1.9–2.8)^a,c,f^	83.45	2.2^–16^
Cholesterol HDL-C ratio	3.9 (3.4–4.3)	3.49 (2.9–4.3)^d^	3.81 (3.3–4.7)^b^	3.58 (3–4.1)^c,f^	14.9	0.0019
**Liver function test**						
ALT/GPT (μIU/mL)	811.3 (19.8–1394)	758 (19.6–1400)	758.4 (52–1329)	817.2 (50.9–1400)	0.604	0.89
AST (mU/mL)	52 (28–74)	52 (34–77)	48.8 (29.4–77.6)	50.5 (33.7–65.8)	0.72	0.86
**Kidney function test**						
Creatinine (μmol/L)	58.5 (49–73.3)	66 (55–79.5)	64.5 (57–76.5)	66 (51–85)	7.07	0.07
eGFR, MDRD formula	98 (87–110.6)	87.7 (74–101.9)^d^	89 (80.5–103)	84 (65.4–108)^c^	15.7	0.0012
**Thyroid function tests**						
TSH (μIU/mL)	2.9 (1.9–3.8)	2.8 (2–4.2)	2.5 (1.7–3.6)	3.6 (2.3–4.7)^a,c,f^	13.6	0.0034
Free T3 (pg/mL)	1.8 (1.5–2)	1.8 (1.5–2.1)	1.9 (1.5–2.2)	1.9 (1.5–2.2)	1.98	0.57
Free T4 (ng/dL)	1.3 (1.1–1.6)	1.3 (1.2–1.6)	1.3 (1.1–1.5)	1.38 (1.2–1.6)	2.8	0.42
**Ion concentration**						
Anion Gap (mmol/L)	14 (12–15)	13 (12–15)	14 (12–15.7)	14 (12–16)	5.91	0.116
Chloride (mmol/L)	105 (104–106)	105 (103–107)	104 (102–106)^b^	104 (102–106)^a,c^	13.19	0.0042
Sodium (mmol/L)	142 (141–143)	142 (141–143)	142 (141–143)	141 (140–142.5)^a,c^	18.5	0.0003
Potassium (mmol/L)	4.4 (4–4.7)	4.4 (4–4.7)	4.25 (4–4.6)	4.5 (4.1–4.8)	6.42	0.093
**Hematological parameters**						
WBC (×10^9/L)	5.7 (4.9–6.8)	5.9 (4.9–7)	6 (5.5–7.3)^e^	6.6 (5.4–7.8)^a,c^	16.4	0.0009
Lymphocytes (abs ×10^9/L)	2 (1.6–2.4)	2 (1.6–2.3)	2 (1.7–2.6)	2.2 (1.8–2.6)^a,c^	11.18	0.011
Monocytes (abs ×10^9/L)	0.41 (0.35–0.49)	0.43 (0.35–0.51)	0.45 (0.37–0.54)^e^	0.47 (0.39–0.59)^a,c^	11.07	0.011
Poly/Neu (abs ×10^9/L)	3.1 (2.6–3.8)	3.2 (2.6–3.9)	3.3 (2.6–4.4)	3.6 (2.9–4.3)^a,c^	9.97	0.019
Eosinophils (abs ×10^9/L)	0.15 (0.12–0.28)	0.17 (0.11–0.27)	0.18 (0.1–0.32)	0.2 (0.13–0.44)^a,c^	8.54	0.034
Basophils (abs ×10^9/L)	0.03 (0.02–0.05)	0.03 (0.02–0.05)	0.03 (0.02–0.05)	0.04 (0.025–0.06)	2.83	0.42
RBC (×10^12/L)	4.5 (4.3–4.9)	4.5 (4.2–4.8)	4.6 (4.3–5)	4.5 (4–4.8)	5.88	0.12
Hematocrit (%)	41.3 (39.6–43.8)	40.9 (38.3–43.8)	41.7 (38.9–44.7)	39.4 (36–42)^a,c,f^	21.9	6.7^–05^
MPV (fL)	10.5 (9.9–10.9)	10.4 (9.9–10.9)	10.7 (10.3–11.1)	10.5 (9.9–11)	7.03	0.071
RBC distribution width (%)	13.2 (12.7–14)	13.3 (12.8–13.9)	13.3 (12.9–14)	13.4 (12.7–14.4)	1.44	0.69
MCH (pg)	30.1 (29.2–30.9)	30 (29–31)	30 (29–31)	29.8 (28.5–30.9)	5.31	0.15
MCHC (g/dl)	32.8 (32.3–33.3)	32.8 (32.3–33.5)	32.7 (32.2–33.6)	33 (32.3–33.7)	1.26	0.74
MCV (fL)	91.9 (89–94)	91.6 (88.8–94)	90.5 (88.8–94)	89.4 (86.5–92.8)^a,c,f^	15.6	0.0013
Platelets (×10^9/L)	233 (206–286)	245 (210–279.7)	244.5 (214–283)	255 (224–297)	5.86	0.12

*Significantdifference (*P* < 0.05) between two groups is indicated by the symbols, ^a^DM vs. Non-DM, ^b^Pre-DM vs. Non-DM, ^c^DM vs. Healthy, ^d^Non-DM vs. Healthy, ^e^Pre-DM vs. Healthy, and ^f^DM vs. Pre-DM. For each measured parameter the result in the table indicate a Median and interquartile range (IQR, 25–75th percentile) or number (%) as appropriate (Poly/Neu, polymorphs/neutrophils).*

### Inflammation in Aging Occurs Independently of Diabetes

**FIGURE 2:**
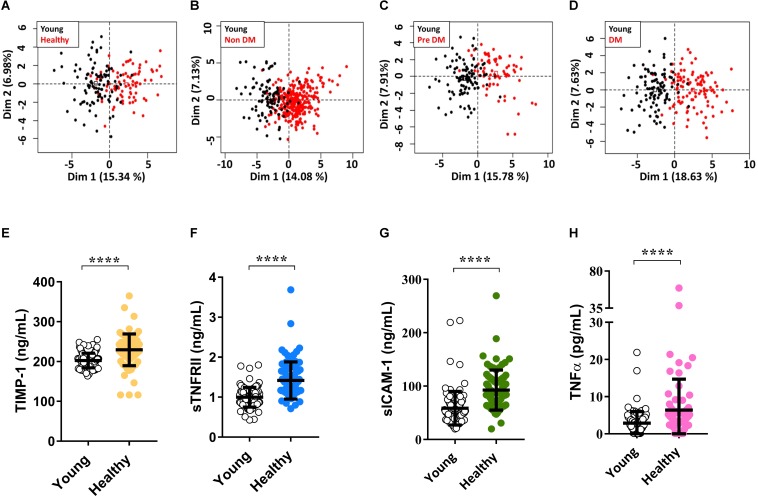
Higher level of the inflammatory molecules with aging. Unbiased analysis of all measured cytokines and chemokines using Principal Component Analysis (PCA) shows a clear separation between Young vs. Healthy **(A)**, Young vs. Non-DM **(B)**, Young vs. Pre DM **(C)**, and Young vs. DM **(D)**. Individual comparison of major cytokines shows a significant difference (*P* < 0.05) between Young vs. healthy for TIMP-1 **(E)**, sTNFRII **(F)**, sICAM-1 **(G)**, and TNFα **(H)**. *P*-values were calculated using non-parametric Mann–Whitney *U*-test and adjusted for Bonferroni multiple test correction (****P < 0.0001).

In order to further investigate the inflammatory process accompanying aging, we first compared the measured inflammatory molecules between the four groups of elderly and young healthy individuals. we measured different molecules including pro-inflammatory molecules like TNFα, IL-6, MIP-1d, anti-inflammatory molecules including IL-10 and TGFb1 (Transforming Growth Factor 1), soluble adhesion molecules like sICAM-1, sE-selectin, sVCAM-1 molecules that regulate extracellular matrix proteins like MMP and TIMP, and molecules indicative of pathological condition like TSH (Thyroid-stimulating hormone). These and other measured molecules give an overview of general health and inflammatory status of an individual. Unbiased analysis of all measured molecules using Principal Component Analysis (PCA) showed separation between young individuals and the four old groups (Figures 2A–D). Whereas no clear separation was found between the four old groups (Supplementary Figures 1A–F). Looking at individual molecules we show a significantly higher (*P* < 0.001) amount of sTNFRII, sICAM-1, and TIMP-1 (Tissue inhibitory Metalloproteinase protein-1) in healthy elderly compared to young individuals. This indicates aging by itself without any comorbidity to be accompanied with an increased level of inflammatory biomarkers (Figures 2E–H). These molecules also correlate with one another much strongly (*P*-value <0.05 for most correlations) in older individuals compared to young individuals (Supplementary Tables 2, 3). This strong correlation of inflammatory biomarkers suggests the formation of a pro-inflammatory network that could associate with loosen balance between pro-inflammatory and anti-inflammatory molecules in disease-free aging (Supplementary Figure 4) (Franceschi et al., 2007).

### Inflammation in DM and Metabolic Syndrome in Aged Individuals

**FIGURE 3:**
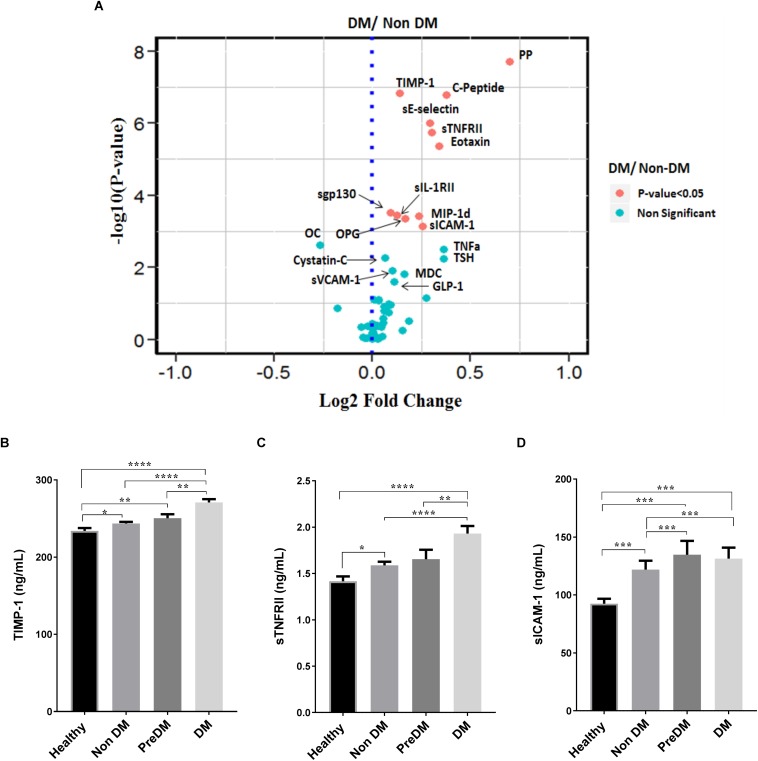
Increased inflammatory molecules in Diabetes individuals. **(A)** A representative Volcano plot comparing all measured molecules between DM and Non-DM individuals, red labeled dots indicate a significant difference (*P* < 0.05) between the two groups. Cytokine comparison between the four groups show significantly high (*P* < 0.05) level in DM individuals for TIMP-1 **(B)**, sTNFRII **(C)**, and sICAM-1 **(D)**. The volcano plot was done by first taking the Fold change (which was calculated by dividing the average value of each molecule in DM and Non-DM individuals) and then using the log2 fold change against the –log10 *P*-value of each molecule. *P*-values were calculated using non-parametric Kruskal–Wallis test and Mann–Whitney *U*-test for comparing for more than two groups and for comparing between two groups, respectively, then adjusted for Bonferroni multiple test correction (ns *P* > 0.05, *P <0.05, **P < 0.01, ***P < 0.001, ****P < 0.0001).

We further compared the level of inflammatory biomarkers between the four groups and find a high level of these molecules in DM individuals. Significantly high level (*P* < 0.05) of metabolic molecules like PP and C-peptide as well as inflammatory biomarkers like TNFα, sTNFRll, sICAM-1, TIMP-1, eotaxin, and sE-selectin were observed in DM group compared to the other groups (Figures 3A–D and Supplementary Figures 2A,B). Pre-DM individuals showed significantly high (*P* < 0.05) level of sICAM-1 when compared to Non-DM and Healthy old individuals (Figure 3D and Supplementary Figure 2D). On the other hand, Healthy old individuals have a significantly lower level (*P* < 0.05) of inflammatory markers compared to the three other groups (Figures 3B–D and Supplementary Figures 2B,C,E) indicating healthy aging as defined by clinical phenotype to be matched by biological (inflammatory) readouts.

**FIGURE 4:**
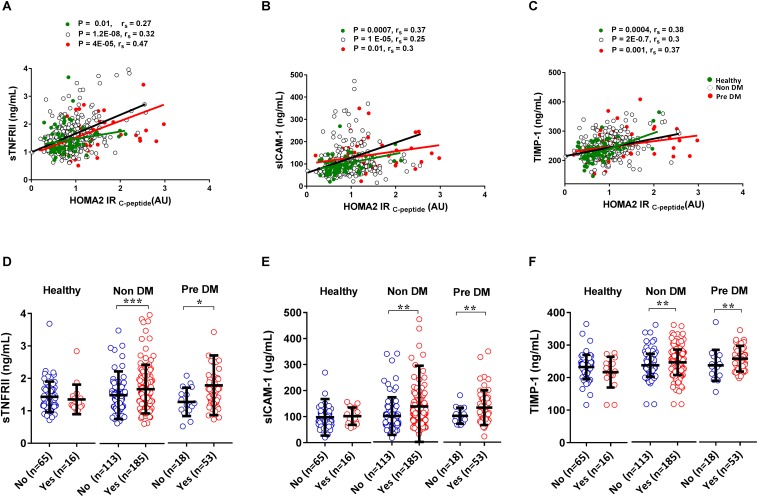
Correlation and association of inflammatory markers with insulin resistance and metabolic syndrome. Inflammatory molecules correlate with an increase in the level of insulin resistance. The green dot indicates Healthy, black circles indicate Non-DM and red dots indicate Pre DM individuals, r indicates Spearman’s rho **(A–C)**. Individuals having metabolic syndrome have a higher level of inflammatory biomarkers **(D–F)**. *P*-values were calculated using non-parametric Mann–Whitney *U*-test for comparing between two groups and spearman correlation test was used to correlate inflammatory molecule with insulin resistance (ns *P* > 0.05, *P <0.05, **P < 0.01, ***P < 0.001).

Metabolic syndrome is a medical condition that predisposes individuals to diabetes and atherosclerotic cardiovascular disease (National Institutes of Health, 2002) whereas insulin resistance leads to a progressive development of diabetes (Taylor, 2012). Here we show that inflammatory biomarkers sTNFRII, sICAM-1 and TIMP-1 significantly (*P* < 0.05) correlate with insulin resistance in Healthy, Non-DM, and Pre-DM individuals indicating early chronic inflammation could be associated with the development of diabetes (Figures 4A–C). By using the updated NCEPATP III criteria (Grundy et al., 2005), we divide each group (except for DM group as almost all have metabolic syndrome, Figure 1C) based on presence or absence of metabolic syndrome. We find that individuals having metabolic syndrome in Non-DM and in Pre-DM group have a significantly higher (*P* < 0.05) level of sTNFRII, sICAM-1, and TIMP-1 (Figures 4D–F). No significant difference was observed in the Healthy group which may be due to low number of individuals having metabolic syndrome in this group. We further divided the five components of metabolic syndrome (abdominal obesity, high Fasting blood glucose, high blood pressure, increased triglyceride, and decrease in HDL cholesterol concentration) and in most cases the accumulation of these components associated with a higher level of sTNFRII, sICAM-1 and TIMP-1 (Table 3).

**TABLE 3:** Inflammatory profile in components of metabolic syndrome.

	Cytokines	No MSC (*n* = 14)	1MSC (*n* = 29)	2MSC (*n* = 20)	3MSC (*n* = 10)	4MSC (*n* = 6)	5MSC	χ^2^	*P*-value
	sICAM-1 (μg/mL)	0.079	0.078	0.101	0.086	0.105	–	8.41	0.08
Healthy	sTNFRII (ng/mL)	1.375	1.252	1.483	1.141	1.378	–	3.75	0.44
	TIMP-1 (μg/mL)	0.235	0.226	0.24	0.212	0.243	–	5.60	0.23

	**Cytokines**	**No MSC (*n* = 11)**	**1MSC (*n* = 42)**	**2MSC (*n* = 58)**	**3MSC (*n* = 91)**	**4MSC (*n* = 93)**	**5MS**	**χ^2^**	***P*-value**

	sICAM-1 (μg/mL)	0.082	0.074	0.082	0.094	0.091	–	9.99	0.04
Non-DM	sTNFRII (ng/mL)	1.2	1.3	1.3	1.4	1.6	–	14.83	0.01
	TIMP-1 (μg/mL)	0.22	0.23	0.23	0.24	0.25	–	10.44	0.03

	**Cytokines**	**No MSC**	**1MSC (*n* = 2)**	**2MSC (*n* = 14)**	**3MSC (*n* = 19)**	**4MSC (*n* = 14)**	**5MSC (*n* = 20)**	**χ^2^**	***P*-value**

	sICAM-1 (μg/mL)	–	0.104	0.095	0.126	0.133	0.107	4	0.41
Pre DM	sTNFRII (μg/mL)	–	0.79	1.28	1.37	1.45	1.72	13	0.01
	TIMP-1 (μg/mL)	–	0.19	0.24	0.24	0.25	0.27	11	0.026

*MSC(Metabolic Syndrome Component) indicated on the table are Abdominal obesity, High Fasting blood glucose, High Blood pressure, Increased triglyceride, and decrease in high-density lipoprotein cholesterol concentration.*

### Metformin Reduces Inflammation and Mortality in DM Patients Compared to Other Monotherapies

**FIGURE 5:**
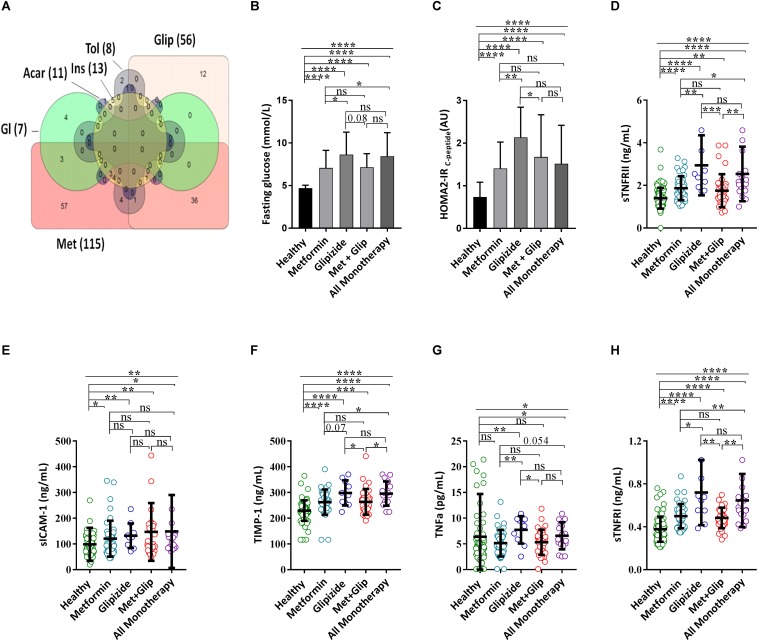
Metformin decrease TNFα associated Systemic Inflammatory molecules. **(A)** Venn diagram [made using online free software (Bioinformatics and Evolutionary Genomics, 2017)] indicates the combination of drug used for a total of 140 diabetes patients (1 patient take a combination of gliclazide and diabetin is not included in the Venn diagram). Comparison of DM patients with different treatments for fasting blood glucose **(B)**, HOMA2-IR_C–peptide_ **(C)**, sTNFRII **(D)**, sICAM-1 **(E)**, TIMP-1 **(F)**, TNFα **(G)**, and sTNFRI **(H)**. *P*-values were calculated using non-parametric Kruskal–Wallis test and Mann–Whitney *U*-test was used for comparing more than two groups and between two groups, respectively (ns *P* > 0.05, *P < 0.05, **P < 0.01,***P < 0.001,****P < 0.0001). Acar, acarbose; Glip, glipizide; Gl, glibenclamide; Ins, Insulin; Tol, tolbutamide; Met, metformin.

The recent interest in metformin as a potential drug to reduce the prevalence of age-related diseases led us to test whether DM patients benefit from the suggested pleiotropic effects of metformin. Metformin monotherapy and glipizide are the two most commonly prescribed monotherapies in our cohort, accounting for 40.4% (57/141) and 8.5% (12/141), respectively, while 25.5% (36/141) of DM patients were under combined metformin and glipizide therapy (Figure 5A). Elderly DM individuals taking metformin monotherapy showed significantly (*P* < 0.05) lower level of sTNFRII, TNFα and of sTNFRI when compared to elderly individuals under glipizide monotherapy and all other monotherapies (Figure 5). Likewise, other molecules including C-peptide, resistin, and C-cystatin also showed significantly lower levels (*P* < 0.05) in diabetes individuals taking metformin monotherapy compared to those that are under Glipizide monotherapy (Supplementary Figure 3). As uncontrolled blood glucose is associated with inflammation we have looked for the level of blood glucose between metformin and other monotherapies and confirmed that individuals under metformin monotherapy have significantly lower (*P* < 0.05) level of fasting blood glucose and HOMA2-IR_C–Peptide_ (Figures 5B,C). We validate the effect of metformin monotherapy over glipizide monotherapy by taking individuals that took the combination of both medications. Diabetes patients taking the combination of metformin and glipizide treatment show a similar trend as those under metformin monotherapy (Figures 5B–D) indicating the anti-inflammatory effects of metformin consumption in elderly DM individuals.

Five-year follow-up mortality data suggest a trend for DM to be associated with higher mortality risks: DM individuals (7.6%) as compared to Healthy (3.3%), Non-DM (4.3%), and pre-DM (6.2%) individuals (Table 4). As our cohort is not powered enough to conclude significantly on this we analyzed the mortality within the DM group. Despite an even smaller number of individuals included in the analysis, the proportion of death that occurred in DM individuals taking metformin monotherapy was 2.4% whereas the proportion of death in DM individuals taking non-metformin monotherapy was 19% [OR = 9.4, CI (1.32–117.4), *P*-value 0.041]. There was no significant difference in the age between the two diabetes treatment groups (Table 4). There was a significant difference (*P* < 0.05) when we compared the age of healthy individuals with diabetes patients taking metformin or other treatments (Table 4). We did not find a significant difference in mortality rate between metformin monotherapy with Healthy, Non-DM, and Pre-DM individuals but there was a significant difference (*P* < 0.05) when we compare the Healthy and Non-DM groups with diabetes patients taking non-metformin monotherapy (Table 4). This suggests metformin to lower the mortality risk of DM individuals to the level of individuals without confirmed diabetes.

**TABLE 4:** Five-years follow up survival data.

Diabetes treatment	Age (year) x (SD)	Alive n (%)	Dead n (%)	OR	95% CI	*P*-value
Metformin (*n* = 41)	68 (7)	40 (97.6)	1 (2.4)	9.4	(1.32–117.4)	0.041
Non Met drugs (*n* = 21)	70 (7.4)	17 (81)	4 (19)			
Metformin (*n* = 41)	68 (7)	40 (97.6)	1 (2.4)	1.37	(0.17–16.5)	0.9
Healthy (*n* = 151)	63 (5.8)	146 (96.7)	5 (3.3)			
Metformin (*n* = 41)	68 (7)	40 (97.6)	1 (2.4)	1.8	(0.29–19.5)	0.9
Non-DM (*n* = 370)	69 (7.8)	354 (95.7)	16 (4.3)			
Metformin (*n* = 41)	68 (7)	40 (97.6)	1 (2.4)	2.6	(0.37–31.7)	0.662
Pre DM (*n* = 81)	68 (7.8)	76 (93.8)	5 (6.2)			
Non-MET Drugs (*n* = 21)	70 (7.4)	17 (81)	4 (19)	0.14	(0.04 – 0.52)	0.014
Healthy (*n* = 151)	63 (5.8)	146 (96.7)	5 (3.3)			
Non-MET Drugs (*n* = 21)	70 (7.4)	17 (81)	4 (19)	0.19	(0.06–0.57)	0.017
Non DM (*n* = 370)	69 (7.8)	354 (95.7)	16 (4.3)			
Non-MET Drugs (*n* = 21)	70 (7.4)	17 (81)	4 (19)	0.28	(0.07–1)	0.083
Pre DM (*n* = 81)	68 (7.8)	76 (93.8)	5 (6.2)			

## Discussion

Inflammation and metabolism are parts of the seven pillars of aging, however, identifying how deregulation in metabolism may alter the inflammatory process remains one of the important questions to be answered in the field (Kennedy et al., 2014). In the present study by including healthy young individuals as control we showed that what is considered as healthy aging (absence of apparent comorbidity) and especially unhealthy aging are accompanied by an increased level of circulating pro-inflammatory molecules (Catania et al., 1997; Bruunsgaard et al., 1999). We further stratified the old cohort into four groups (Healthy, Non-DM, Pre-DM, and DM) and showed that the presence of comorbidities itself were associated with an even higher burden of inflammation (Butcher et al., 2014).

The main inflammatory biomarkers shown to increase with age TIMP1, sTNFRII and sICAM1 are even present at a higher level in diabetes patients. The role of the sTNFRI/II is not fully understood but is known to block TNFα by forming a complex and competing with membrane TNFRs (Van Zee et al., 1992). Thus, the level of the soluble receptors indicate the level or degree of activation of the TNF pathway (Diez-Ruiz et al., 1995) and imbalance between the soluble receptors and TNFα may result in different clinical outcomes (Aderka, 1996). Similarly to ours, previous studies have reported an increase in the level of sTNFRll in diabetes patients compared to controls (Plomgaard et al., 2007) and being predictive of the most important complications of diabetes including nephropathies (Sharma et al., 2015; Carlsson et al., 2016) and heart failures (Ping et al., 2017). sICAM-1 represent a circulating form of ICAM-1 (CD54) that bind to lymphocyte function-associated antigen (LFA-1) and was associated with different pathological conditions (Witkowska and Borawska, 2004). The presence of sICAM-1 in most of the significant comparisons we performed suggests that inflammation in diabetes is also linked to some form of endothelial dysfunction. TIMP-1 is a broad spectrum natural inhibitor of different matrix metalloproteinases (MMPs) including MMP-9 which are involved in modulating the extracellular matrix (Brew and Nagase, 2010). A previous study has shown that levels of TIMP-1 increase with age and also increases the risk of heart disease (Sundstrom et al., 2004). TNFα induces the release of sICAM-1 in a MMP-9 dependent manner (Tsai et al., 2014). These interactions between TNFα, sICAM, MMPs and TIMP-1 could explain our findings concerning the concomitant increased level of these molecules in elderly individuals as well in individuals with diabetes. Furthermore, we have also observed a strong correlation between levels of TIMP1, sTNFRl, sTNFRll, TNFa and sICAM1 in elderly individuals compared to young individuals.

As most these molecules are part of the TNFα system it could indicate the formation of a strong pro-inflammatory network at an older age. As diabetes was also associated with TNF family upregulation it is likely that different sources of dysregulation may lead to ultimately modulate the TNF pathway. One study has shown an increased level of TNFα, sTNFRl, and sTNFRll associated with frailty and decrease in mobility in elderly women (Langmann et al., 2017). A similar finding was observed in elderly individuals with dementia where an increased level of TNFα molecule was positively correlated with sTNFRII, IL-6, and C-Reactive Protein (Bruunsgaard et al., 1999; Maggio et al., 2006; Rea et al., 2018). We even demonstrate the co-existence of this pro-TNF inflammatory state in individuals with metabolic syndrome and insulin resistance. Taken together with other studies this collectively suggests the shedding of TNFR and ICAM in some of the major comorbidities and syndrome of older adults (Fernandez-Real et al., 2002; Winkler et al., 2002). Moreover, an increased sICAM-1 activity was shown to correlate with an increased activity of sTNFRII and TNFα (Straczkowski et al., 2002). We also showed that individuals with metabolic syndrome have a higher level of TIMP1 and similar results were reported elsewhere (Papazoglou et al., 2010; Hopps et al., 2013). These data suggest the importance to consider the presence or absence of metabolic syndrome when interpreting data relating to inflammaging.

The United Kingdom Prospective Diabetes Study has shown obese diabetes patients under metformin monotherapy to have a lower rate of all-cause mortality as well as diabetes-related mortality when compared to those treated with other monotherapy or undergoing conventional treatment (UK Prospective Diabetes Study Group, 1998). Similarly, newly diagnosed diabetes patients that take metformin monotherapy or in combination with sulfonylurea show a reduced mortality rate due to all-causes as well as cardiovascular associated death compared to diabetes patients under sulfonylurea treatment (Johnson et al., 2002). In line with previous reports, another important finding of our study is the low level of mortality rate in individuals taking metformin monotherapy compared to other diabetes treatment. It is of note that the study population consisted of elderly individuals exhibiting other comorbidities. Still, metformin in diabetes elderly individuals show significant effect in this pilot study. This can be partly explained by the observed effect of metformin in lowering inflammation. In this context, previous studies have shown an association between all-cause mortality and baseline sTNFRI levels (Luna et al., 2013) and increased level of both sTNFRI and sTNFRII predict the risk of mortality due to chronic kidney disease and cardiovascular events (Neirynck et al., 2015; Carlsson et al., 2016; Gohda et al., 2017). TIMP1 was also shown to be a good predictor of all-cause mortality in a 10 year follow up study (LaRocca et al., 2017). Altogether, this provides more supporting data for the potential repurposing of metformin to reducing the burden of age-related diseases. This could be achieved by targeting inflammation as one of the pleiotropic effects of metformin.

Some factors influencing inflammation and the concept of inflammaging have not been tested in this study. One typical example could be the presence of persistent chronic infection such as cytomegalovirus which alters immune cell homeostasis and inflammation (Fulop et al., 2013). Another limitation of our study is the sample size of DM individuals taking the various treatments. Additionally, our cohort was separated in young and elderly individuals while evidence show the role of biological age in driving the organism to differential clinical trajectories (Belsky et al., 2015). Biological age is often related to physical functions and basic clinical/biological markers of physiological functions and in our study we have stratified individuals based on diabetes but also comorbidities. We observed that the heterogeneity in inflammatory marker levels could be reduced by such stratification. The pro-inflammatory phenotype was more pronounced in DM individuals under other therapy than metformin. Inflammation was incremental in the following sequence: Young → Healthy elderly → Non-DM = DM with metformin → Pre-DM → DM without metformin. Studies are required to validate the impact of metformin on mortality and identify the mechanisms behind this effect. We propose inflammation as one the processes regulated by metformin through a better control of glucose.

In summary, our study showed the importance of stratification by clinical phenotypes to understand the contribution and role of inflammation in old age. The further stratification by drug usage suggests metformin to be a potential mean of intervention for achieving healthspan by decreasing the inflammatory burden associated with the various age-related pathological conditions. As metformin was not able to restore inflammatory molecules to the level found in young individuals, it is suggested that age-related inflammation, i.e., inflammaging, cannot be targeted by the pathways linked to metformin consumption. It is also plausible that the aging organism sustains the low-grade inflammation, despite metformin or other drugs, as it may have beneficial effects. While chronic inflammation in pathological conditions has been shown to be often detrimental to the individual, more efforts should made to investigate whether inflammaging, as an adaptation to avoid maladaptation of other systems. Understanding the pleiotropic effects of other drugs widely used in the elderly population could help better understand and target inflammation, this applies to cholesterol lowering drugs and anti-hypertension drugs. The same applies to promising compounds and associated pathways with an anti-aging potential such as rapamycin (mTOR) and nicotinamide riboside (Sirtuins).

## Ethics Statement

The study was approved by the National Universityof Singapore Institutional Review Board, and all participants provided written informed consent.

## Author Contributions

AT contributed to the conceptualization of the study, analyzed the data, interpreted the data, and wrote the manuscript. KS, EM, CX, JC, CT, and WH measured and organized the Luminex data. OC set-up and helped with the Luminex experiments. SH supervised the study. EC measured the Fructosamine. TF gave intellectual advice on the analysis, interpretation of the data, and contributed to writing the manuscript. TN and MN coordinated and collected the data from the SLAS-2 cohort. AL conceptualized the study, supported the data analysis, supervised the study, interpreted the data, and contributed to writing the manuscript.

## Conflict of Interest Statement

AT, OC, KS, EM, CX, JC, CT, WH, SH, and AL were employed by A*STAR. The remaining authors declare that the research was conducted in the absence of any commercial or financial relationships that could be construed as a potential conflict of interest. The handling Editor is currently co-organizing a Research Topic with one of the authors, AL, and confirms the absence of any other collaboration.

## Abbreviations

Acar: acarbose
AGP: alpha-1 acid glycoprotein
Gl: glibenclamide
Glip: glipizide
GLP-1: glucagon-like peptide-1
HDL: high-density lipoprotein
Ins: insulin
LDL: low-density lipoprotein
MCH: mean corpuscular hemoglobin
MCHC: mean corpuscular hemoglobin concentration
MCV: mean corpuscular volume
MDC: macrophage-derived chemokine
Met: metformin
MIP-1d: macrophage inflammatory protein-1 delta
MM9: matrix metalloproteinase 9
MPV: mean platelet volume
OC: osteocalcin
OPG: osteoprotegerin
PDGF-AA: platelet derived growth factor AA
Poly/Neu: polymorphs/neutrophils
PP: pancreatic polypeptide
RBC: red blood cell
SAA: serum amyloid A
sE-selectin: soluble E-selectin
sgp130: soluble glycol protein 130
sICAM-1: soluble intracellular adhesion molecule-1
sIL-1RII: soluble interleukin-1 receptor II
sTNFRI: soluble tumor necrosis factor receptor I
sTNFRII: soluble tumor necrosis factor receptor II
sVCAM-1: soluble vascular cell adhesion molecule-1
TIMP-1: tissue inhibitory of matrix metalloprotease
TNFα: tumor necrosis factor-alpha
Tol: tolbutamide
TSH: thyroid stimulating hormone
WBC: white blood cell.
