# Supplementary figures and images for: Metformin Monotherapy Downregulates Diabetes-Associated Inflammatory Status and Impacts on Mortality
Source: Front Physiol. 2019 May 21;10:572. doi: 10.3389/fphys.2019.00572 (PMC6537753; doi:10.3389/fphys.2019.00572)

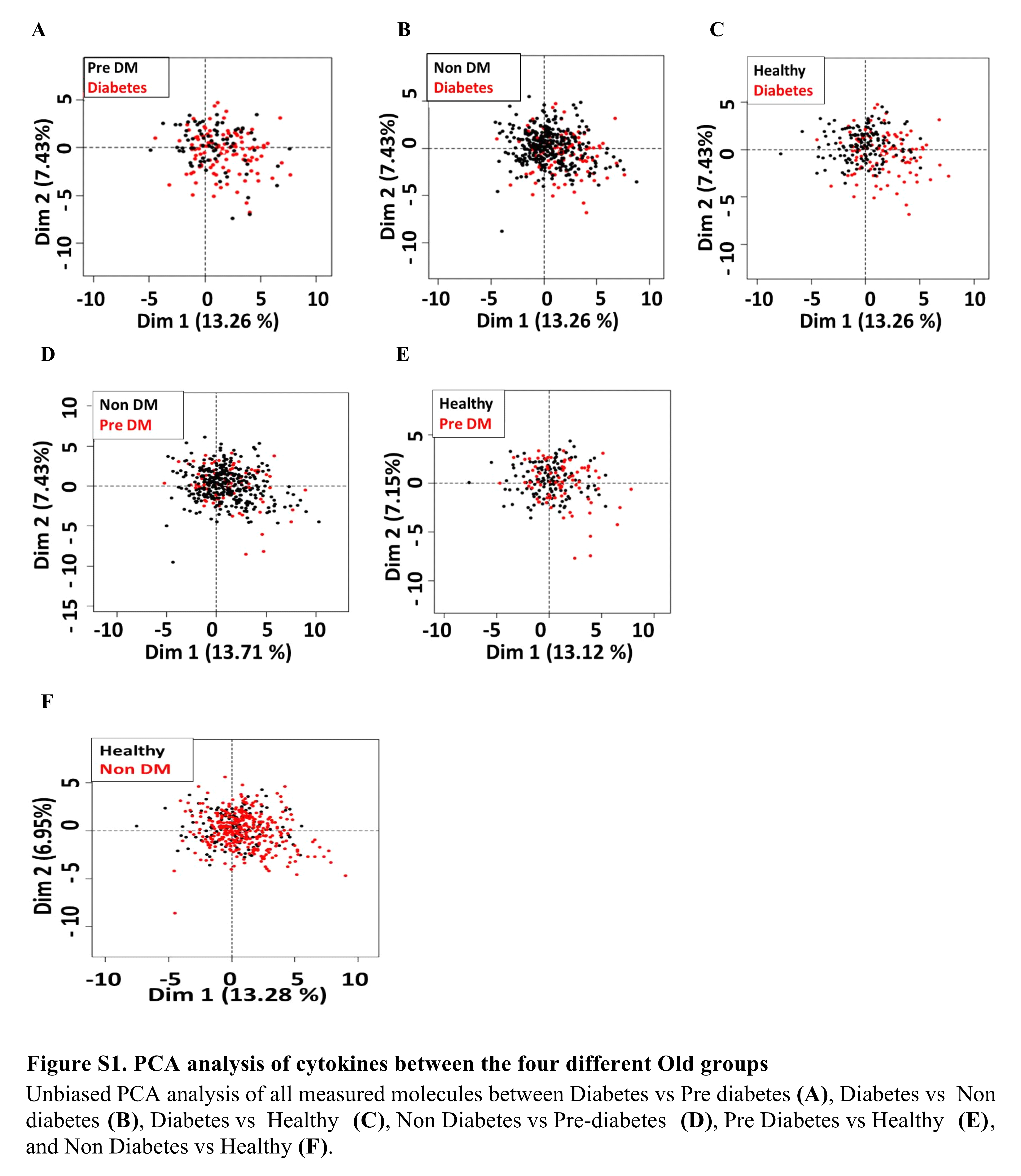

Supplement: Supplementary file 1 [file Image_1.TIF]

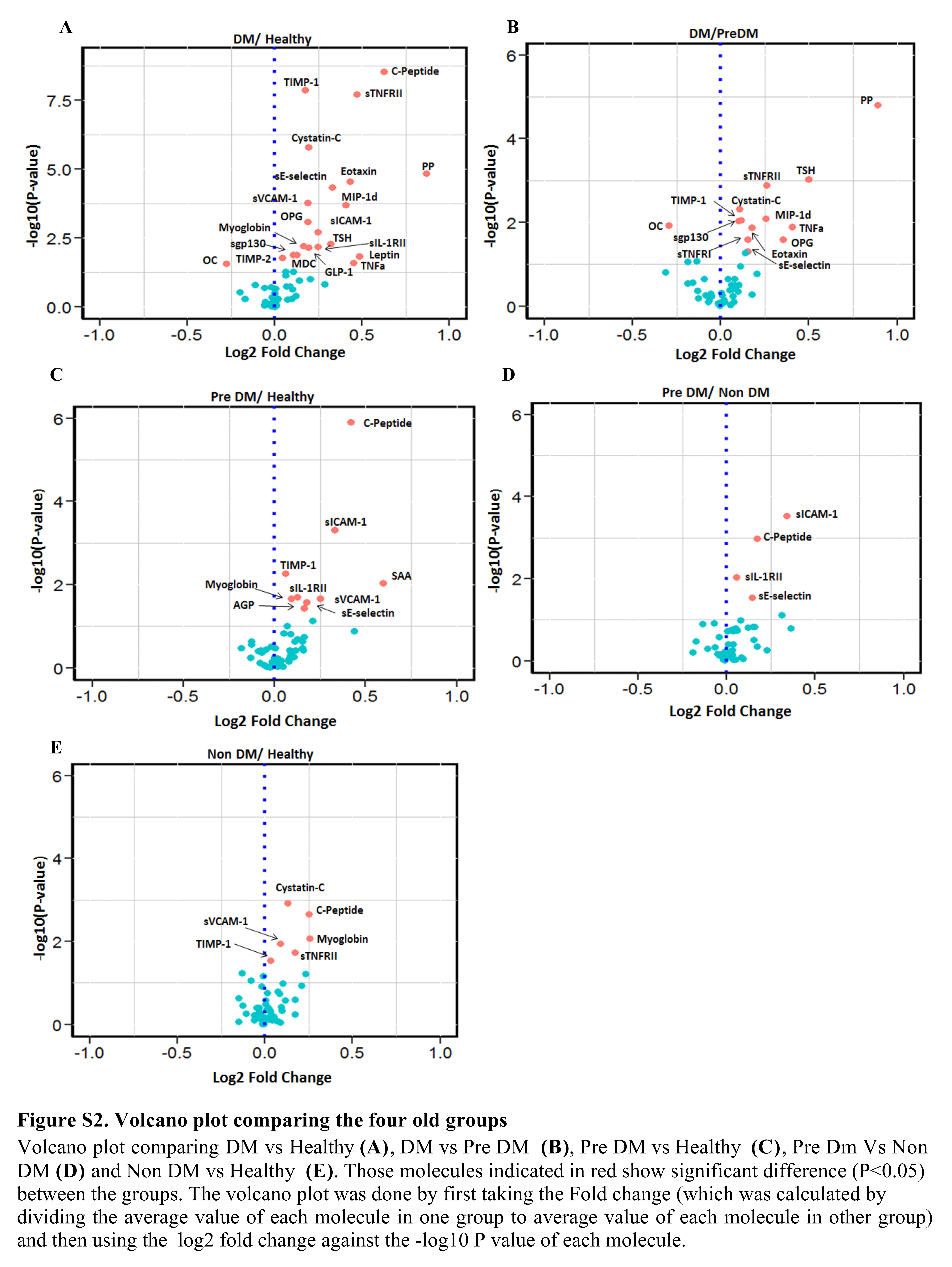

Supplement: Supplementary file 2 [file Image_2.TIF]

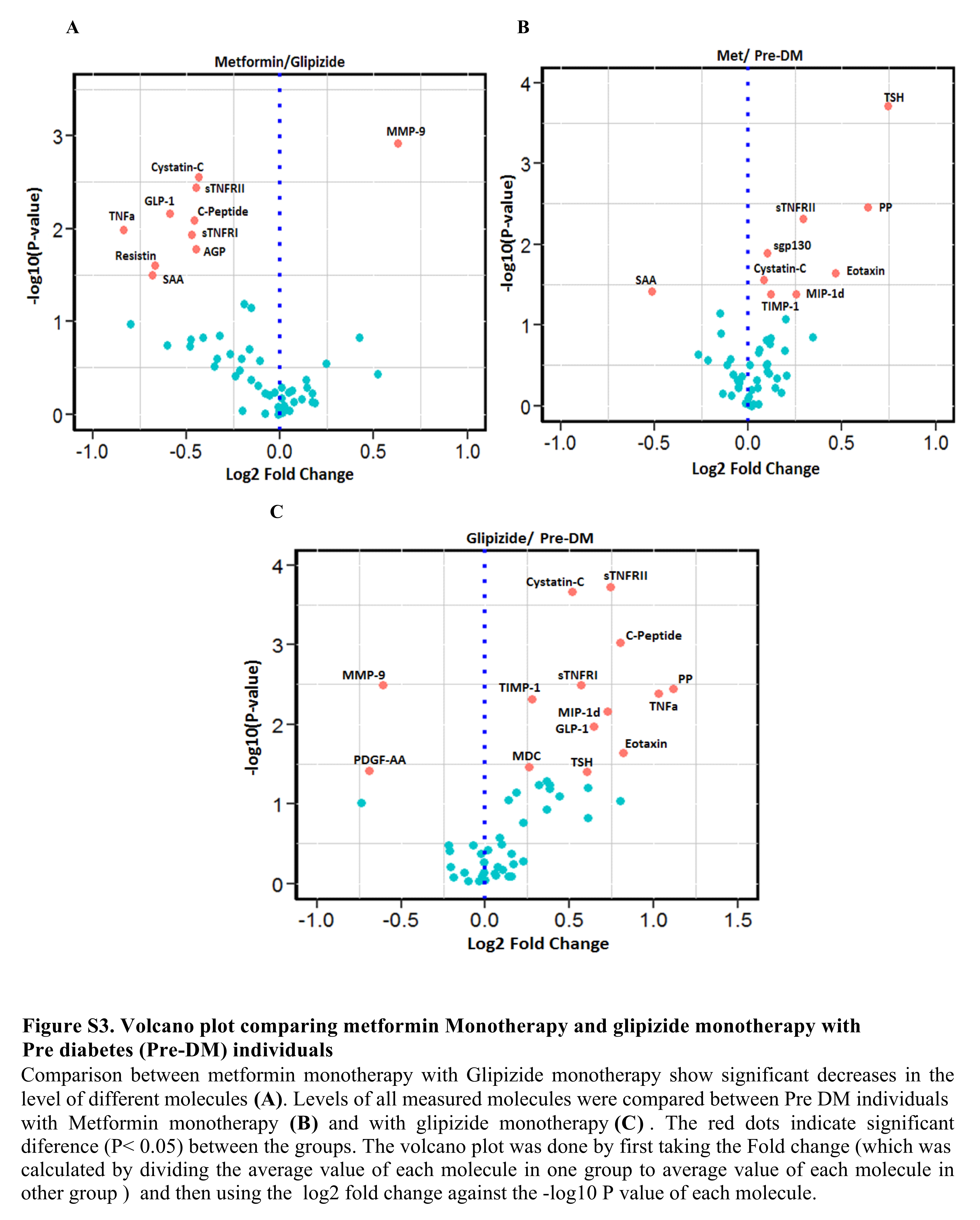

Supplement: Supplementary file 3 [file Image_3.TIF]

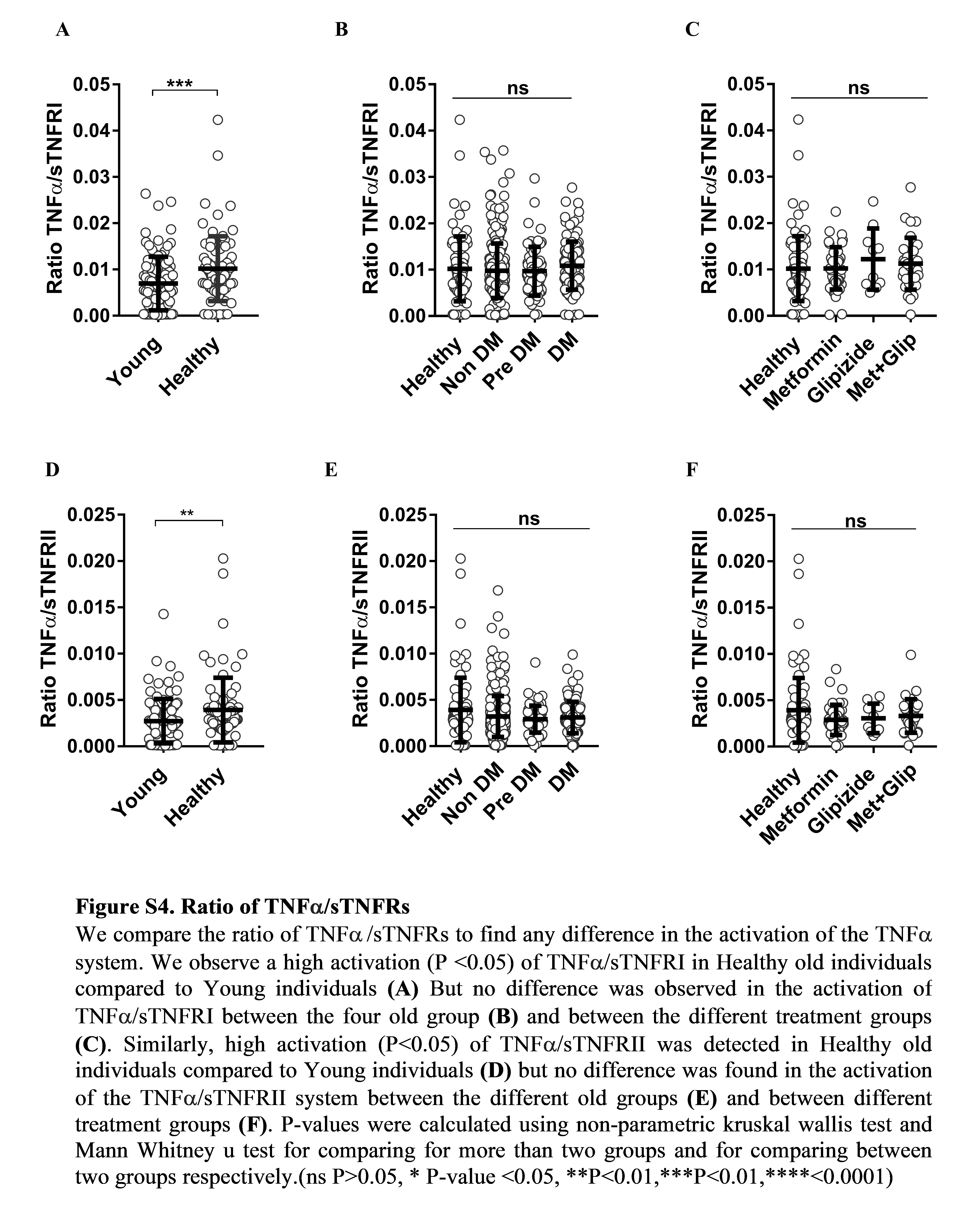

Supplement: Supplementary file 4 [file Image_4.TIF]
