# Supplementary material for: Metformin Monotherapy Downregulates Diabetes-Associated Inflammatory Status and Impacts on Mortality
Source: Front Physiol. 2019 May 21;10:572. doi: 10.3389/fphys.2019.00572 (PMC6537753; doi:10.3389/fphys.2019.00572)
Supplement: Supplementary file 5 [file Table_1.DOC]

**Table S1. Prevalence of disease between DM, Non DM and Pre DM groups**

| Type of Comorbidities | | Non Diabetes  (n=299) | Pre Diabetes  (n=70) | Diabetes  (n=108) | X-squared | p-value |
| --- | --- | --- | --- | --- | --- | --- |
| High Cholesterol | **No** | 123 (41.1) | 45 (64.3) ^b^ | 19 (17.6) ^a,c^ | 40.108 | 1.95^-09^ |
|  | **Yes** | 176 (58.9) | 25 (35.7) | 89 (82.4) |  |  |
| Hypertension | **No** | 111 (37.1) | 46 (65.7) ^b^ | 28 (25.9) ^a,c^ | 29.251 | 4.45^-07^ |
|  | **Yes** | 188 (62.9) | 24 (34.3) | 80 (74.1) |  |  |
| Depression | **No** | 293 (98) | 64 (91.4) ^b^ | 107 (99.1) ^c^ | 10.926 | 0.004242 |
|  | **Yes** | 6 (2) | 6 (8.6) | 1 (0.9) |  |  |
| Heart Disease | **No** | 269 (90) | 65 (92.9) | 89 (82.4) ^a^ | 5.9423 | 0.05124 |
|  | **Yes** | 30 (10) | 5 (7.1) | 19 (17.6) |  |  |
| Kidney Disease | **No** | 293 (98) | 68 (98) | 100 (92.6)^a^ | 7.2013 | 0.02731 |
|  | **Yes** | 6 (2) | 2 (2) | 8 (7.4) |  |  |
| Joint Pain | **No** | 265 (88.6) | 68 (97.1) | 96 (88.9) | 4.7127 | 0.09477 |
|  | **Yes** | 34 (11.4) | 2 (2.9) | 12 (11.1) |  |  |
| Osteoporosis | **No** | 267 (89.3) | 66 (94.3) | 93 (86.1) | 2.9724 | 0.2262 |
|  | **Yes** | 32 (10.7) | 4 (5.7) | 15 (13.9) |  |  |
| Anaemia | **No** | 295 (98.7) | 70 (100) | 108 (100) |  | 0.6182 |
|  | **Yes** | 4 (1.3) | 0 (0) | 0 (0) |  |  |
| Arthritis | **No** | 291(97.3) | 68 (97.1) | 103 (95.4) | 1.0169 | 0.6014 |
|  | **Yes** | 8 (2.7) | 2 (2.9) | 5 (4.6) |  |  |
| Asthma | **No** | 284 (95) | 68 (97.1) | 102 (94.4) | 0.7402 | 0.6907 |
|  | **Yes** | 15 (5) | 2 (2.9) | 6 (5.6) |  |  |
| Nerve Disease | **No** | 274 (91.6) | 67 (95.7) | 101 (93.5) | 1.5361 | 0.4639 |
|  | **Yes** | 25 (8.4) | 3 (4.3) | 7 (6.5) |  |  |
| Gastric Problem | **No** | 254 (84.9) | 58 (82.9) | 90 (83.3) | 0.28122 | 0.8688 |
|  | **Yes** | 45 (15.1) | 12 (17.1) | 18 (16.7) |  |  |
| Thyroid Disease | **No** | 289 (96.7) | 67 (95.7) | 106 (98.1) |  | 0.5224 |
|  | **Yes** | 10 (3.3) | 3 (4.3) | 2 (1.9) |  |  |
| Eye Disease | **No** | 288 (96.3) | 69 (98.6) | 106 (98.1) |  | 0.5709 |
|  | **Yes** | 11 (3.7) | 1 (1.4) | 2 (1.9) |  |  |
| Breathiness | **No** | 297 (99.3) | 70 (100) | 106 (98.1) |  | 0.3201 |
|  | **Yes** | 2 (0.7) | 0 (0) | 2 (1.9) |  |  |
| Skin Disease | **No** | 292 (97.7) | 70 (100) | 107 (99.1) |  | 0.4476 |
|  | **Yes** | 7 (2.3) | 0 (0) | 1 (0.9) |  |  |
| Prostate Cancer | **No** | 296 (99) | 68 (97.1) | 107 (99.1) |  | 0.3966 |
|  | **Yes** | 3 (1) | 2 (2.9) | 1 (0.9) |  |  |

*Significant difference (P<0.05) between two groups is indicated by the symbols, ^a^ DM VS Non-DM , ^b^ Pre-DM and ^c^ DM VS Pre- DM. P value was calculated using either chi-square test or Fisher exact test as appropriate.
