# Supplementary material for: Metformin Monotherapy Downregulates Diabetes-Associated Inflammatory Status and Impacts on Mortality
Source: Front Physiol. 2019 May 21;10:572. doi: 10.3389/fphys.2019.00572 (PMC6537753; doi:10.3389/fphys.2019.00572)
Supplement: Supplementary file 6 [file Table_2.DOC]

**Table S2. Unbiased Spearman’s correlation of major inflammatory biomarkers in the SLAS cohort**

|  | sICAM-1 (ng/mL) | | TNFα (pg/mL) | | TIMP-1 (ng/mL) | | | sTNFRI (ng/mL) | | | | sTNFRII (ng/mL) | |
| --- | --- | --- | --- | --- | --- | --- | --- | --- | --- | --- | --- | --- | --- |
|  | P-value | r_s_ | P-value | r_s_ | P-value | r_s_ | P-value | | | r_s_ | | P-value | r_s_ |
| sICAM-1(ng/mL) |  |  |  |  |  |  |  | |  | | |  |  |
| TNFα (pg/mL) | 1.1^-07^ | 0.22 |  |  |  |  |  | |  | | |  |  |
| TIMP-1 (ng/mL) | 2.2^-16^ | 0.35 | 2.2^-16^ | 0.40 |  |  |  | |  | | |  |  |
| sTNFRI (ng/mL) | 7.6^-06^ | 0.19 | 2.2^-16^ | 0.49 | 2.2^-16^ | 0.40 |  | | | |  |  |  |
| sTNFRII (ng/mL) | 2.8^-14^ | 0.31 | 2.2^-16^ | 0.44 | 2.2-^16^ | 0.64 | 2.2^-16^ | | | | 0.51 |  |  |

* r_s_ indicates Spearman rho
