# Supplementary material for: Metformin Monotherapy Downregulates Diabetes-Associated Inflammatory Status and Impacts on Mortality
Source: Front Physiol. 2019 May 21;10:572. doi: 10.3389/fphys.2019.00572 (PMC6537753; doi:10.3389/fphys.2019.00572)
Supplement: Supplementary file 7 [file Table_3.DOC]

**Table S3. Unbiased Spearman correlation of major Inflammatory biomarkers in young participants**

|  | sICAM-1 (ng/mL) | | TNFα (pg/mL) | | TIMP-1 (pg/mL) | | | sTNFRI (pg/mL) | | | | sTNFRII (pg/mL) | |
| --- | --- | --- | --- | --- | --- | --- | --- | --- | --- | --- | --- | --- | --- |
|  | P-value | r_s_ | P-value | r_s_ | P-value | r_s_ | P-value | | | r_s_ | | P-value | r_s_ |
| sICAM-1(ng/mL) |  |  |  |  |  |  |  | |  | | |  |  |
| TNFα (pg/mL) | 0.23 | 0.12 |  |  |  |  |  | |  | | |  |  |
| TIMP-1 (pg/mL) | 0.74 | -0.03 | 0.91 | 0.01 |  |  |  | |  | | |  |  |
| sTNFRI (pg/mL) | 0.89 | -0.01 | 0.72 | 0.04 | 0.0829 | 0.174 |  | | | |  |  |  |
| sTNFRII (pg/mL) | 0.19 | -0.13 | 0.05 | 0.20 | 0.0003 | 0.357 | 0.0006 | | | | 0.34 |  |  |

*r_s_ indicates Spearman rho
